# Dystrophin‐Deficient Muscular Dystrophy in a Jack Russell Terrier With a Large Deletion in the Canine *DMD* Gene

**DOI:** 10.1002/vms3.71110

**Published:** 2026-07-25

**Authors:** Emilie Royaux, G. Diane Shelton, Vidhya Jagannathan, Tosso Leeb, Michaela Drögemüller

**Affiliations:** ^1^ Davies Veterinary Specialists Hitchin UK; ^2^ Comparative Neuromuscular Laboratory, Department of Pathology University of California San Diego San Diego California USA; ^3^ Institute of Genetics, Vetsuisse Faculty University of Bern Bern Switzerland

**Keywords:** canine, Duchenne, dystrophinopathy, immunohistochemistry, precision medicine

## Abstract

A 6‐month‐old male Jack Russell Terrier presented with failure to thrive, lethargy, muscle atrophy, hypersalivation, and exercise intolerance. Similar signs were reported for the only other male puppy in the litter that died prematurely, whereas all the female puppies developed normally. An electromyography (EMG) test revealed abnormal electrical activity, such as complex repetitive discharges and fibrillation potentials. Cryosections of skeletal muscles revealed degenerative and regenerative myopathy with calcific deposits, consistent with a form of muscular dystrophy. Immunohistochemical testing demonstrated a lack of dystrophin, alongside upregulation of utrophin, and a subsequent reduction in dystrophin‐associated proteins, which is consistent with dystrophin‐deficient muscular dystrophy. Whole genome sequencing revealed a complex structural variant including a deletion of approximately 25.5 kb encompassing exons 8 and 9 of the *DMD* gene, which was absent in more than 1750 unrelated control genomes. This loss‐of‐function variant most likely accounts for the clinical presentation. Subsequent PCR testing revealed the presence of the wild‐type allele in both parents and a normal female littermate. Amplification of the mutant allele could not be achieved. The mutant allele was likely transmitted from the mother to the index case and the presumed affected male littermate. It remains unresolved whether the mutant allele was transmitted over several generations in the maternal line or arose by a *de novo* mutation event in the germline of the mother. In conclusion, we describe a partial deletion of the *DMD* gene in a Jack Russell Terrier with a Duchenne‐like muscular dystrophy phenotype due to non‐functional dystrophin.

## Graphical abstract

A 6‐month‐old male Jack Russell Terrier presenting with muscle wasting and lethargy was diagnosed with Duchenne‐like muscular dystrophy based on clinical, electromyographic, histopathological, and immunohistochemical evidence of dystrophin deficiency. Whole genome sequencing identified a novel 25.5 kb deletion encompassing exons 8 and 9 of the *DMD* gene, resulting in non‐functional dystrophin. This likely maternally inherited loss‐of‐function allele explains the severe phenotype and expands the spectrum of canine *DMD*‐associated variants.

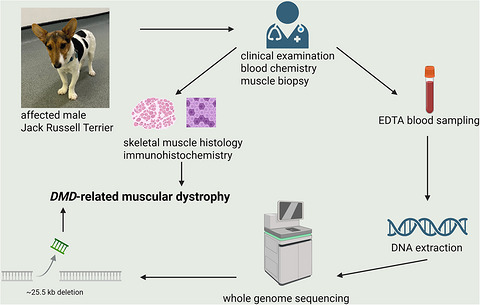

## 1 Background

Muscular dystrophy refers to a group of inherited myopathies with specific defects affecting various skeletal muscle proteins. The most common form of muscular dystrophy in humans and dogs is associated with the *DMD* gene encoding the sarcolemmal protein dystrophin. The structural components of dystrophin mediate a mechanical function that stabilizes and links the muscle cell membrane and the cytoskeleton. *DMD* is located on the X chromosome, and dystrophin‐deficient muscular dystrophy is inherited as an X‐linked recessive trait. Defective alleles are often transmitted from unaffected carrier females to their hemizygous mutant affected sons. X‐linked dystrophin‐deficient muscular dystrophy is the veterinary analogue of Duchenne and Becker muscular dystrophy of humans. In Duchenne muscular dystrophy, functioning dystrophin is completely absent in the muscle, while in Becker muscular dystrophy, some residual dystrophin function is preserved, but not enough for completely normal function. The two conditions differ in their severity, age of onset, and rate of progression. The signs of Becker muscular dystrophy are usually milder.

Common clinical signs in dystrophin‐deficient dogs include progressive muscle atrophy, weakness, stiff gait, plantigrade stance due to tarsal joint contracture, exercise intolerance, excessive salivation due to pharyngeal dysfunction, weak bark, kyphosis that progresses to lordosis, and hypertrophy of the muscles of the base of the tongue and selected limb muscles. Signs of muscular dystrophy are typically seen early in life and progress as the dog ages. The serum creatine kinase (CK) activity is markedly increased (i.e., >20000 IU/L) in dogs diagnosed with the X‐linked dystrophin‐deficient muscular dystrophy (Bergman et al. 2002; Jones et al. 2004; Kornegay et al. 1988). This is not a pathognomonic finding as similar increases in CK have been described in dogs with necrotizing myopathies (Shelton 2004; Wells et al. 2009). Electromyography (EMG) typically reveals complex repetitive discharges (CRD) and myotonic discharges (Valentine et al. 1989). These changes can also be seen in cases of chronic denervation, non‐dystrophic congenital myotonias or other chronic myopathies (Kornegay et al. 1988). A diagnosis is based on histopathology of muscle biopsies with demonstration of a dystrophic pathological phenotype. Typical findings on histopathology are degeneration (myonecrosis) and regeneration of the muscle fibers, phagocytosis, endomysial fibrosis, and myofiber mineralization (Vandevelde et al. 2012). Dystrophin‐deficient muscular dystrophy can be confirmed by using antibodies against dystrophin protein by immunohistochemistry and immunoblotting and/or genetic analysis. Specific disease causing variants in the *DMD* gene have been identified in various breeds of dog (Online Mendelian Inheritance in Animals [OMIA] #001081‐9615) such as in the Australian Labradoodle (Shrader et al. 2018), Border Collie (Mata López et al. 2018; Van Poucke et al. 2024), Brittany Spaniel (Shelton et al. 2023), Cavalier King Charles Spaniel (Nghiem et al. 2017; Walmsley et al. 2010), Cocker Spaniel (Kornegay et al. 2012), French Bulldog (Shelton et al. 2023), German Shorthaired Pointer (Schatzberg et al. 1999), Golden Retriever (Sharp et al. 1992), Jack Russell Terrier (Brunetti et al. 2020), Japanese Spitz (Atencia‐Fernandez et al. 2015), Labrador Retriever (Barthélémy et al. 2020; Kornegay et al. 2012; Shelton et al. 2021; Shelton et al. 2022; Smith et al. 2007), Miniature Poodle (Sánchez et al. 2018), Norfolk Terrier (Jenkins and Forman 2015), Pembroke Welsh Corgi (Smith et al. 2011), Rottweiler (Winand et al. 1994), Tibetan Terrier (Kornegay et al. 2012), Entlebucher Mountain Dog (Schwarz et al. 2024), and Shiba Inu (Mcleay et al. 2025). Extensive research has been performed towards the treatment of muscular dystrophy, but so far, no curative treatment has been identified (Kornegay 2017). Corticosteroids, used at anti‐inflammatory dosages, are known to improve the phenotype or at least to slow the disease evolution (Markham et al. 2008; Merlini et al. 2012; Pradhan et al. 2006; Wong and Christopher 2002). There is active research with gene therapy in canine models of this myopathy. The severity of dysfunction is variable, but the overall prognosis is poor.

The objectives of this case report were (1) to describe the clinical and pathological features of a young Jack Russell Terrier with dystrophin‐deficient muscular dystrophy, and (2) to identify the putative genetic cause using a wholegenome sequencing (WGS) approach.

## 2 Materials and Methods

### 2.1 Ethics Statement

Our study did not require regulatory or institutional ethical approval because it was not experimental, but rather part of a clinical and pathological veterinary diagnostic evaluation. All animals in the study were examined with the consent of their owners and were treated according to ethical standards.

### 2.2 Signalment and History

The study included one affected dog (the case animal), its normal female littermate, and their sire and dam, and genomic data of 1757 dog control genomes. Blood samples were processed in the VET_GEN_BERN collection of the Vetsuisse Biobank.

The case was a 6‐month‐old male Jack Russell Terrier presented with failure to thrive, lethargy, muscle atrophy, hypersalivation, and exercise intolerance. There was no evidence of parental consanguinity within at least two generations. Neither the father nor the mother had any other litters. One additional male offspring, born in the same litter as the present case, was reported to have similar signs and died prematurely. All four of the female littermates developed normally.

### 2.3 Clinical Examination

A complete clinical examination of the case was performed, including a general clinical examination and a neurological clinical assessment. Additionally, complete blood count (CBC) and plasma biochemistry were performed.

### 2.4 Histopathology

Skeletal muscle biopsies for histological evaluation were collected from the right *M. biceps femoris* and *M. triceps brachii* of the case under general inhalational anaesthesia. Unfixed chilled biopsies were flash frozen in isopentane precooled in liquid nitrogen, then sections cut (8 µm) and stained or reacted using a standard panel of histochemical stains and reactions (Dubowitz et al. 2021). The fixed biopsy was evaluated in routine paraffin sections stained with hematoxylin and eosin (H&E).

### 2.5 Immunohistochemistry

To characterize a specific form of muscular dystrophy, cryosections (8 µm) were cut from the *M. biceps femoris* and *M. triceps brachii* and an archived control muscle and stained for indirect immunofluorescence as previously described (Guo et al. 2010). Several monoclonal or polyclonal antibodies were used including those against the rod (1:100, NCL‐DYS1) and carboxy‐terminus (1:100, NCL‐DYS2) of dystrophin, and against utrophin (1:20, NCL‐DRP2), developmental myosin heavy chain (dMHC1:20, NCL‐dMHC), α‐sarcoglycan (1:200, gift of Eva Engvall) (Liu and Engvall 1999), β‐dystroglycan (1:100, NCL, bDG), laminin α2 (gift of Eva Engvall, 4F11, direct apply) (Leivo and Engvall 1988), and collagen VI (gift of Eva Engvall, 3G7, direct apply) (Hessle and Engvall 1984).

### 2.6 DNA Extraction

Genomic DNA was isolated from EDTA blood of the case, the littermate, and both parents using a Maxwell RSC48 instrument and the Maxwell RSC Whole Blood DNA Kit (Promega, Dübendorf, Switzerland).

### 2.7 Whole Genome Sequencing and Variant Calling

A TruSeq PCR‐free Illumina DNA library with an insert size of ∼400 bp was prepared from the genomic DNA of the affected dog. This was then sequenced at 22.3x coverage on an Illumina NovaSeq 6000 instrument. The reads were mapped to the UU_Cfam_GSD_1.0 reference genome assembly, as previously described (Jagannathan et al. 2019). To identify variants associated with the described phenotype, the genotype of the sequenced case was compared with 1757 genetically diverse dog genomes (Table S1). All candidate variants were visually inspected using the Integrative Genomics Viewer (IGV) (Robinson et al. 2017).

### 2.8 PCR‐Based Validation

To confirm the presence of the deletion, a PCR approach was chosen. A set of three primers F_del_ 5′‐TGAAAGCCTTGCCATCTCAC‐3′, F_wt_ 5′‐TCAATGGCTGGACAAGAGTT‐3′ and R 5′‐ACAGGCTTTGCAAGATACAGT‐3′ was designed. The primer F_del_ had a binding site in the deleted region, while F_wt_ and R had binding sites distal to the deletion. The reverse primer was used in combination with both different forward primers independently. PCR products were amplified from genomic DNA using AmpliTaq Gold 360 Master Mix (Thermo Fisher Scientific, Reinach, Switzerland) and visualized by fragment length analysis using a 5200 Fragment Analyzer System (Agilent, Santa Clara, CA, USA).

## 3 Results

### 3.1 Clinical Presentation

A 6‐month‐old, male entire, Jack Russell Terrier was presented for failure to thrive, lethargy, muscle atrophy, hypersalivation, and exercise intolerance. The dog was born in a litter of six puppies, which consisted of 4 female and 2 male puppies. Both male puppies were significantly smaller compared with the female puppies from birth. The other male puppy died when only a few months old from a suspected aspiration pneumonia. Blood tests in the surviving male puppy performed by the referring veterinary surgeon revealed a significant increase in CK activity (47990 U/L, reference range 20.0‐225.0). Serology for *Toxoplasma gondii* and *Neospora caninum* was not compatible with an active infection. General physical examination revealed a soft left basilar heart murmur. The neurological examination was normal except for generalized muscle atrophy (video 1: https://youtu.be/rx1Dcd8bAy8). Based on the signalment, history, physical, and neurological examination, a myopathy was suspected. The CK activity was measured again and had further increased to 136390 U/L (0‐350). Cardiac ultrasound revealed no significant changes. Radiographs of the chest revealed no obvious megaoesophagus or aspiration pneumonia. An airway inspection was done and revealed hypertrophy of the muscles of the base of the tongue. EMG was performed on multiple muscles of the left thoracic and pelvic limbs. EMG changes were most obvious in the proximal appendicular muscles (video 2: https://youtu.be/msigol‐oQ58). Abnormal electrical activity, such as complex repetitive discharges and fibrillation potentials, was seen.

Taken together, the clinical findings were consistent with a form of muscular dystrophy.

### 3.2 Pathological Features

Cryosections from the right triceps and biceps muscles revealed a degenerative and regenerative myopathy with calcific deposits consistent with a form of muscular dystrophy (Figure 1).

**FIGURE 1:**
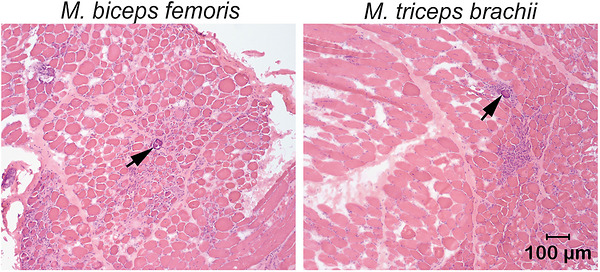
Histological staining of skeletal muscles. H&E‐stained cryosections of the *M. biceps femoris* and *M. triceps brachii* from the Jack Russell Terrier with dystrophin‐deficient muscular dystrophy. Variability in myofiber size was present with numerous degenerating and regenerating fibers, scattered myofibers with internal nuclei and calcific deposits (arrows). These changes are consistent with a dystrophic phenotype. Bar in triceps muscle image = 100 µm for both muscles.

Immunostaining of muscle cryosections using several monoclonal antibodies against dystrophy‐related proteins revealed an absence of dystrophin protein with upregulation of utrophin (Figure 2), and secondary reduction of the dystrophin‐associated proteins compatible with dystrophin‐deficient muscular dystrophy.

**FIGURE 2:**
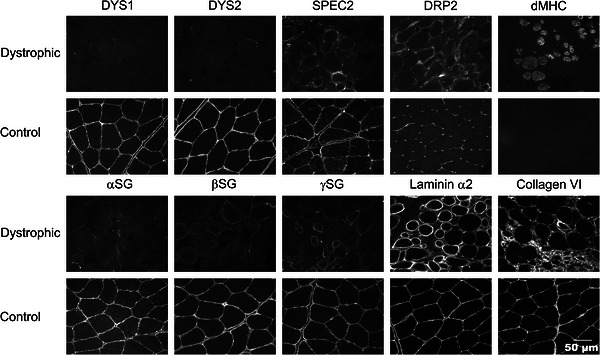
Immunofluorescent staining of skeletal muscle cryosections. Immunofluorescence staining of cryosections from the *M. biceps femoris* of the dystrophic dog and archived control canine limb muscle using monoclonal antibodies against dystrophin (DYS1 and DYS2), against spectrin for membrane integrity (SPEC2), against utrophin (DRP2), against dMHC for regenerating fibers, against α, β, and γ‐sarcoglycans (αSG, βSG, and γSG), against laminin α2, and collagen VI. Staining with both DYS1 and DYS2 was absent or markedly reduced with upregulation of utrophin on the muscle sarcolemma. Staining for the three sarcoglycans was moderately reduced. Staining for laminin α2 was normal in appearance, and mild endomysial fibrosis was shown in the staining for collagen VI. Bar in lower right image = 50 µm for all images.

Based on the results of histopathology and immunostaining, a diagnosis of dystrophin‐deficient muscular dystrophy was made. Treatment with corticosteroids (prednisolone 5 mg every other day) was started. The dog remained relatively stable over 1 year of follow‐up but was finally euthanized at 1 year and 9 months of age due to increased difficulty in drinking and swallowing.

### 3.3 Genetic Findings

Whole genome sequencing of the affected dog revealed a large hemizygous structural variant involving a ∼25.5 kb deletion in the region of the *DMD* gene (Figure 3A,B). The proximal breakpoint was located in a repetitive region with low mapping quality of the short reads. Our data did not contain any reads spanning the deletion breakpoint, suggesting that the structural variant is more complex than a simple deletion (e.g., comprising an additional insertion, Figure 3C,D). From now on, we will refer to this complex structural variant, which has not been fully resolved, as a deletion, since it involves the deletion of coding exons 8 and 9 (Figure 3C). The tentative formal variant designation is NC_049260.1:g.28,018,818_28,044,266del or NC_049260.1(NM_001003343.1):c.650‐12906_963+11013del. Assuming unchanged splicing of the remaining exons, the deletion removes 314 coding nucleotides and was predicted to cause a frameshift, resulting in the introduction of a premature stop codon, NP_001003343.1:p. (Asp217AlafsTer5).

**FIGURE 3:**
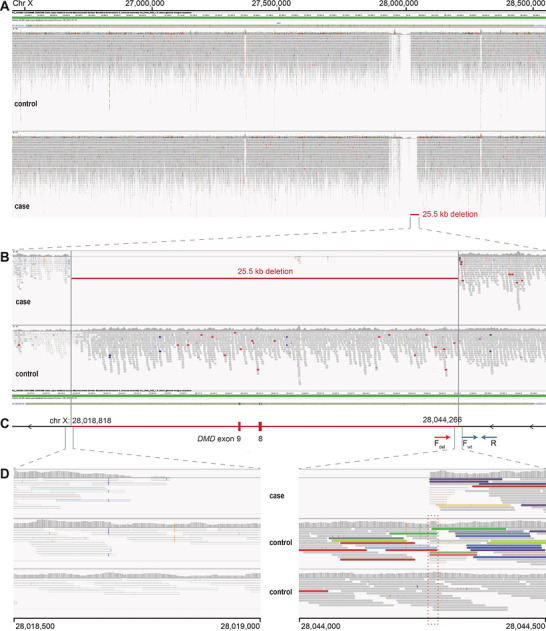
Multi‐exon deletion within the *DMD* gene in the affected Jack Russell Terrier. **(A)** Overview (IGV screenshot) across the entire canine *DMD* gene from the whole genome sequence data in comparison to a control dog. The 25.5 kb deletion is indicated. It is located immediately adjacent to a region with high repeat content that cannot be reliably sequenced with short‐read data. **(B)** Details of the 25.5 kb‐sized deletion with breakpoints. **(C)** The deletion spans *DMD* exons 8 and 9 (highlighted in red) and part of the flanking introns. **(D)** Details of the breakpoints. The proximal breakpoint is in a repetitive region with low mapping quality of sequencing reads. The distal breakpoint is located at the position of an independent polymorphic ∼6.4 kb LINE‐1 insertion in dogs. This insertion is present in the affected dog and the first control dog shown, but absent from the second control dog shown and the UU_Cfam_GSD_1.0 reference genome. The repetitive nature of the flanking sequences prevented successful amplification of the mutant allele.

No other protein‐changing private variants were detected in the *DMD* gene. The deletion was absent from 1757 dogs, including a similarly affected Jack Russell Terrier from Italy (Brunetti et al. 2020). PCR analysis revealed the presence of the wild‐type allele in all available family members except the affected dog (Figure 4). The repetitive structure of the sequence at the breakpoints did not allow the successful amplification of a specific product for the mutant allele. We were therefore unable to determine whether the two females in the family were heterozygous carriers or clear of the deletion.

**FIGURE 4:**
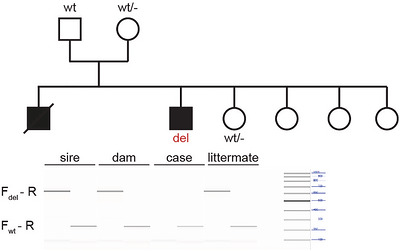
Pedigree of the family and PCR‐based determination of the presence or absence of the *DMD* wild‐type allele. Genotypes are indicated for all four dogs for which samples were available. The genotypes of the four other littermates remain unknown. The early deceased male puppy is indicated by a strikethrough symbol. We assume that this puppy was also affected, but the puppy was not examined in detail. In the genotype designation of hemizygous male dogs, only one allele is indicated. In the female dogs, the presence of the wild‐type allele was confirmed, but zygosity and carrier status are unknown. The unknown second allele is indicated by a hyphen. Note that the F_del_/R amplicon is only absent in the affected dog (location of primers is shown in Figure 3C).

### 3.4 Discussion

This case report describes the clinical signs, diagnostic findings, and genetic analysis of a young Jack Russell Terrier with dystrophin‐deficient muscular dystrophy.

This condition is characterized by a novel structural variant including the partial deletion of the *DMD* gene, which differs from the genetic variant previously described in a similarly affected Jack Russell Terrier in Italy (Brunetti et al. 2020). Assuming that the splicing of the remaining exons is unaltered in the affected dog, deletion of exons 8 and 9 introduces a premature stop codon, which is expected to result in nonsense‐mediated decay of the mutant transcript. This is consistent with absent DMD protein staining in immunohistochemistry and confirms that this variant leads to a complete loss of function.

We speculate that the male littermate that showed similar signs of muscular dystrophy and died early in life also received the same mutant *DMD* allele from its dam. Unfortunately, no sample material was available from the deceased puppy to confirm its genotype. It remains unresolved whether the mutant allele was transmitted over several generations in the maternal line or arose by a *de novo* mutation event in the germline of the mother.

The 25.5 kb deletion likely resulted from an unequal crossing‐over event between homologous LINE‐1 sequences that flank exons 8 and 9 in a head‐to‐tail orientation. The LINE‐1 sequence on the distal side of the deletion is not present in the UU_Cfam_GSD_1.0 reference genome. It represents an older structural variant that is independent of the pathogenic 25.5 kb deletion. Both alleles at this structural variant (with and without the LINE‐1 insertion) are common in dogs of different breeds.

Other spontaneous *DMD* variants detected in several dog breeds encompass small deletions and insertions, nonsense and splicing variants, as well as structural variants, including inversions and duplications. These observations provide a comprehensive foundation for investigating the genetic variability of the disease (Shelton et al. 2023; Online Mendelian Inheritance in Animals [OMIA] #001081‐9615). This case highlights the value of integrating clinical, pathology, and genetics to improve understanding of rare diseases in dogs.

### 3.5 Conclusion

In this study, we describe the clinical, histopathological, immunohistochemical and molecular genetic details of a Jack Russell Terrier with dystrophin‐deficient muscular dystrophy. Taken together, the observed complex structural variant with a large genomic deletion of ∼25.5 kb, which comprises two coding exons of the *DMD* gene, most likely caused the experimentally confirmed lack of protein expression, resulting in the phenotype in the investigated dog.

## Author Contributions

**Emilie Royaux**: conceptualization, investigation, methodology, visualization, writing – original draft preparation, writing – review and editing. **G. Diane Shelton**: investigation, methodology, visualization, writing – original draft preparation, writing – review and editing. **Vidhya Jagannathan**: data curation, writing – review and editing. **Tosso Leeb**: writing – review and editing. **Michaela Drögemüller**: conceptualization, investigation, methodology, visualization, writing – original draft preparation, writing – review and editing, and project administration. All authors have read and agreed to the published version of the manuscript.

## Funding Information

The authors have nothing to report.

## Ethics Statement

Our study did not require regulatory or institutional ethical approval because it was not experimental, but rather part of a clinical and pathological veterinary diagnostic evaluation. All animals in the study were examined with the consent of their owners and were treated according to ethical standards.

## Conflicts of Interest

The authors declare no conflicts of interest.

## Supporting information




**Supplementary File 1**: vms371110‐sup‐0001‐TableS1.xlsx

## Data Availability

The data that support the findings of this study are openly available in ENA at https://www.ebi.ac.uk/ena/, reference number SAMEA114382026.
